# Impact of COVID-19 Measures on Discharge Planning and Continuity of Integrated Care in the Community for Older Patients in Singapore

**DOI:** 10.5334/ijic.6416

**Published:** 2022-05-13

**Authors:** Sungwon Yoon, Jiahui Mo, Zhui Ying Lim, Si Yinn Lu, Sher Guan Low, Bangyu Xu, Yu Xian Loo, Chee Wai Koh, Lai Yee Kong, Rachel Marie Towle, Su Fee Lim, Chuen Seng Tan, Yu Heng Kwan, Lian Leng Low

**Affiliations:** 1 Health Services and Systems Research, Duke-NUS Medical School, Singapore; 2 Centre for Population Health Research and Implementation, SingHealth Regional Health System, Singapore; 3 Duke-NUS Medical School, Singapore; 4 Population Health & Integrated Care Office, Singapore General Hospital, Singapore; 5 Research and Translational Innovation Office, SingHealth Community Hospitals, Singapore; 6 Post-Acute and Continuing Care, Sengkang Community Hospital, Singapore; 7 Post-Acute and Continuing Care, Outram Community Hospital, Singapore; 8 Medical Social Services, Outram Community Hospital, Singapore; 9 Medical Social Services, Sengkang Community Hospital, Singapore; 10 Specialty Nursing, Population Health & Integrated Care Office, Regional Health System, Singapore; 11 RHS Community Nursing, Population health & Integrated Care Office, Singapore General Hospital, Singapore; 12 Saw Swee Hock School of Public Health, National University of Singapore and National University Health System, Singapore; 13 Department of Pharmacy, National University of Singapore, Singapore; 14 Department of Family Medicine and Continuing Care, Singapore General Hospital, Singapore; 15 SingHealth Duke-NUS Family Medicine Academic Clinical Program, Singapore

**Keywords:** COVID-19, discharge planning, integrated care, older patients, care continuity

## Abstract

**Introduction:**

The COVID-19 pandemic affects the process of care transition for patients with underlying chronic conditions. This study aims to explore the impact of the pandemic measures on discharge planning and continuum of care for vulnerable older patients from multi-stakeholder perspectives.

**Methods:**

We conducted focus group discussions and individual interviews with healthcare workers, community partners, government officials and family caregivers in Singapore. All interviews were audio-recorded, transcribed verbatim and thematically analysed.

**Results:**

A total of 53 individuals participated in the study. Discharge planning and care continuity in the community were affected primarily by the limited step-down care options and remote assessment of discharge needs. Participants felt a need to revisit the decision of ‘essential’ community services through engagement of all stakeholders to enhance care community.

To improve better care transition, participants suggested the need for clearer communication of guidelines, improved intersectoral collaboration, shared responsibility of patient care through community engagement and employment of novel models of care.

**Conclusion:**

The pandemic measures generated challenges of safe discharge of patients and care continuity in the community. Findings shed light on the need to proactively assess care pathways and catalyse novel models to improve care transition beyond the pandemic.

## Introduction

Since the initial cases of COVID-19 were detected in December 2019, the pandemic has spread to more than 200 countries, affecting millions worldwide [1], including Singapore. Singapore is a densely populated multi-ethnic city-state, and its position as a global travel hub makes it vulnerable to the importation and spread of infectious diseases. Despite efforts to contain the virus through vigorous contact tracing and strict isolation, mass outbreaks in foreign worker dormitories occurred in April 2020 [2]. This spurred the government’s decision to impose a two-month lockdown to limit the community spread of the virus.

During the lockdown, many services deemed ‘non-essential’ were either curtailed or ceased, including services that supported older adults living in the community with long term care needs [3,4]. Essential services were defined as those, if not provided, that would result in significant or rapid deterioration of the patient’s medical condition, and potentially threaten their health and wellbeing. For example, residential and home-based community services such as nursing homes were allowed to function while services such as day care centres, senior care centres, day rehabilitation centres, psychiatric rehabilitation centres, day hospices, home therapy and medical escort services were closed [3,4]. In addition, healthcare workers were deployed to serve on the frontline [5], resulting in manpower strains and potential disruption of essential services.

Disruption of community services can lead to suboptimal discharge planning and care discontinuity. Literature suggests that in addition to manpower shortage, poor communication among healthcare professionals [6] and inadequate community services are some of the key factors contributing to delayed discharge [7] and affecting the care continuum for patients [8]. Recent studies also illustrate that decreased access to community services disproportionately affected older patients with complex health and social needs, which may create considerable demands on emergency services and heavy cost implications to the health system [9,10]. Increased mental health risks resulting from the lack of community services have also been observed; sense of loneliness and lack of perceived social support in the community were found to be associated with geriatric depression [11]. Depression increases the risk of subsequent chronic illnesses [12] and suicidal ideations [13].

To date, there have been extensive studies evaluating the symptoms of COVID-19 patients as well as their management in acute care settings [14]. Studies have also shown fewer hospital visits and admissions amongst older non-COVID patients in acute care setting [15,16,17], with the mean length of hospital stay shorter compared to pre-COVID years [15,17]. In regards to the delivery of patient care in the community, increased aggressiveness or violence among psychiatric patients was observed following reduced non-compulsory treatment and consultation [18]. Literature also found elevated anxiety levels in older cancer patients who faced difficulties seeking care in the community [19]. Although these studies provided important insights into the experience of non-COVID patients being adversely affected by the pandemic, limited research exists as to how the pandemic and its associated measures affected discharge planning and seamless care for older patients from rehabilitation and sub-acute wards of community hospitals during this critical period of transition.

To fill the gap, this study aims to explore the impact of COVID-19 pandemic measures and the subsequent curtailment of community care services on discharge planning and continuity of integrated care from the perspectives of multi-stakeholders, including caregivers of patients, healthcare workers (HCWs), community partners and policymakers. We also sought to understand what stakeholders perceived as essential community services amidst the pandemic and their suggestions for providing a more seamless transition of care.

## Methods

### Setting

This study was undertaken within the SingHealth cluster. SingHealth is the largest public healthcare cluster in Singapore serving more than 50% of the country’s population by providing a comprehensive range of medical care through a network of five national specialty centres, four tertiary hospitals, a network of eight public primary care clinics and three community hospitals [20]. To ensure patient-centric integrated care, the cluster provides a range of step-down care services particularly for older patients who require dedicated long-term care. Agency for Integrated Care (AIC), an independent entity under Ministry of Health (MOH), works with health and community care partners in developing and coordinating the delivery of aged care services [21].

### Participant recruitment and data collection

We recruited caregivers of patients, HCWs, community partners and government officials during the period between July 2020 and February 2021. We recruited family caregivers of patients aged 65 years and above who were discharged from rehabilitation and sub-acute wards of two major community hospitals within three months. Patients were identified from the administrative databases by two of the study team members (medical doctors). The study team also identified a list of HCWs of varying occupations and duration of service involved in discharge planning in two community hospitals and a restructured hospital through publicly available institutional websites and the team’s professional network. Additionally, government officials from major government bodies (i.e., MOH) were approached based on their roles in integrated community care services. Participants were contacted by telephone (patients/caregivers) or via email (HCWs and officials), and the study purpose and confidential and anonymous participation were explained. A purposive sampling technique was used to obtain a diverse range of views until data saturation was achieved.

A semi-structured interview guide was developed with open-ended questions and pilot tested. Topics included i) perceptions of non-essential services that were halted during lockdown; ii) impacts of COVID-19 measures on discharge planning, iii) continuity of care and self-management in patients admitted to community hospitals; and iv) suggestions to improve patient care and outcomes in times of similar public health emergencies. Consented individuals were invited to participate in an individual interview or Focus Group Discussion (FGD) subject to availability. Each FGD comprised 2 to 4 participants. An experienced facilitator (ZYL, SL) trained in qualitative research conducted the interviews remotely via videoconferencing tools with the presence of one note-taker (JM). Each FGD and individual interview lasted about 90 to 120 minutes and 45 to 60 minutes, respectively and audio recorded. Verbal informed consent was recorded prior to the interviews. The findings presented in this paper does not reflect the views of the funding agency.

### Data analysis

All audio recordings were transcribed verbatim. NVivo 12 was used for thematic analysis based on a grounded theory approach [22]. We coded inductively line by line after each completed interview/FGD, and this further directed and refined the interview guide for subsequent interviews. Coding categories were developed from open coding to an analytical coding of the text until a series of interlinking themes and patterns were elicited. Two independent coders (JM, SYL) from the study team performed the analysis. Discrepancies were resolved through consecutive rounds of discussion and recoding among study team members. Outstanding coding disputes were simultaneously examined by another team member (SY). This paper also followed the Consolidated Criteria for Reporting Qualitative Research (COREQ) checklist to ensure comprehensive and transparent reporting of the research [23]. This study was approved by the SingHealth Centralised Institutional Review Board (Ref: 2020/2374).

## Results

### Participant characteristics

We conducted 13 FGDs and 17 individual interviews involving 53 participants: 32 HCWs, 5 community partners, 6 government officials and 10 patient’s caregivers. Thematic saturation was reached after 22 interviews/FGDs with HCWs and government officials and 8 interviews with patient’s caregivers, respectively. No HCWs, government officials and community partners declined participation whilst out of 68 patients and their caregivers approached, 58 declined. Major reasons for rejection included worsening health condition of patients and finding remote consent and interviews troublesome. For HCWs, community partners and government officials, the median years of experience in practice were 11 years, with a majority being Chinese (69.8%) and female (90%). For patients and their caregivers, the vast majority were Chinese (90%) and female (88%). Caregiver interviews were conducted in the presence or absence of patients. The median age of patients being cared by caregivers was 82.5 years old (Table 1).

**Table 1:** Participant characteristics (N = 53).


	HCWS, COMMUNITY PARTNERS AND GOVERNMENT OFFICIALS (N = 43)	CAREGIVERS (N = 10)
	
	N (%)	N (%)

Number of Interviews	**22**	**8**

FGDs	12	1

IDIs	10	7

Profession		

Nurse	16 (37.2)	–

Doctor	7 (16.3)	–

Medical Social Worker	6 (14.0)	–

Physiotherapist/Occupational Therapist	3 (7.0)	–

Community Partner	5 (11.6)	–

Government Official	6 (14.0)	–

Median years of experience in practice (range)	11 (0–29)	**–**

Median years of caregiving (range)	**–**	**1.5 (0.3–10)**

Median age of patient (range)	**–**	**82.5 (67–89)**

Gender		

Male	4 (9.3)	2 (20)

Female	39 (90.7)	8 (80)

Ethnicity		

Chinese	30 (69.8)	9 (90)

Malay	9 (20.9)	1 (10)

Indian	4 (9.3)	0 (0)


### Impacts of COVID-19 on discharge planning and continuity of care in the community

The main themes surrounding the impacts of the pandemic on discharge planning and care continuity were broadly classified into three domains: patient and caregiver related, HCW related, and government policy and health services related (Table 2).

**Table 2:** Summary of themes and subthemes.


	THEME/DOMAIN	SUBTHEME

Impact of COVID-19 on discharge planning and continuity of care in the community	Patient and caregiver related	Rejection of services by family members due to fear of COVID-19 infection

		Limited digital literacy to adopt telehealth and tele-rehab as an alternative service

		Inadequate communication with patients and their caregivers for post-discharge care

	Healthcare worker related	Fear of infection during home visit leading to uncertainty in decision making

		Manpower shortage due to workforce redeployment

	Government policy and health services related	Limited stepdown care options available

		Alternative services perceived to have limited benefits

		Insufficient communication of COVID-19 restriction measures

Perceptions of essential services for care integration during lockdown	Services felt to be essential for continuum of care despite lockdown	Day Rehab/Day Care centres: to maintain wellbeing of patients and reduce caregiver burden

		Cluster Support and case management: to ensure continuous monitoring of older adults in community

		Medical Escort and Transport services: to ensure continuity of treatment

		Home modification: to facilitate discharge and secure safe home environment

	Curtailment or closure of services deemed justifiable	Prevention of COVID-19 transmission as top priority

Ways to improve seamless transition of care	Patient and caregiver related	Empowering patients through improved digital literacy

		Greater interpersonal interactions to enhance mental wellbeing of patients and caregivers

	Healthcare worker related	Change in outlook – adaptability and positivity

		Improving intersectoral communication and multidisciplinary collaboration among care providers

	Government policy and health services related	Fostering communication between authority and healthcare professionals and caregivers

		Revisiting definitions of “essential” services

		Adapting service models to prepare for times of crisis

		Integrated platform to streamline services


Under the *patient and caregiver related domain*, a rejection of community services over the fear of COVID-19 infection by patients and family members was a prominent theme that affected timely discharge planning. Accounts from both HCW and caregiver participants reflected a general sense of reluctance to accept integrated home and day care services among family caregivers because there were concerns that homecare workers were “bringing the virus to the house”. To improve care continuum, technology-enabled services such as tele-consultation or tele-rehabilitation were adopted in the community to provide a smooth transition of care. However, many HCW participants found the use of telehealth to be challenging for older adults due to limited digital literacy, hearing impairment and inability to adequately describe one’s symptoms, limiting the seamless delivery of care in the community.

*“When we provide tele-consult, it is difficult to assess, because some assessment requires face to face review and when the resident is unable to tell specifically about their symptoms, for example, like leg swelling or any skin colour, this kind of things they are unable to describe. Then it’s difficult to assess whether their condition is still maintaining or is deteriorating.” (#P013, nurse, F)*

Under the *HCW-related domain*, a recurrent theme was the difficulty of assessing caregivers’ level of support needs post-discharge over the phone due to safe distancing measures, which inevitably generated a lack of confidence in decision-making for discharge.

*“Well, I think communication, there’s beyond just voice, there’s non-verbal communication. So going on face to face I can also note who is the wife, who I’m speaking to, is she big size, small size, can easily take care of her husband or not, you know. I can also see from their non-verbal cues how committed they are. And face to face also allows the opportunity to address some of their concerns on the ground and I can liaise, and I can always pass them brochures, which over the phone is very difficult.” (#P022, doctor, F)*

In addition, community partners and HCWs recounted how they had felt vulnerable while conducting home visits owing to the limited infection prevention measures and equipment at the onset of the pandemic. Although fear of COVID-19 exposure did not seem to have a large impact on seamless delivery of care in the community, it certainly created a dilemma for HCWs and community partners to streamline decisions and focus on tasks of patient care.

*“In the beginning when things were not stable, colleagues were quite worried about conducting home visits. We were told that we should not go out unnecessarily, yet we were the ones entering patients’ home and some family had many family members. So certain colleagues had concerns whether it would be safe to continue conducting home visits or not. Then at that point of course, mask wearing was not compulsory, so probably less reassuring as well.” (#P023, doctor, F)*

Manpower shortage also appeared to create strains on care provision; participants noted that redeployment of manpower to COVID-related work reduced the manpower to facilitate discharge procedures and support patients in the community.

*“Because most of our community nurses have been called back to be deployed to hospital, the cases that left behind, you know those community nurses that remain behind have to take over the cases to make sure that these residents are well cared for in the community.” (#P002, nurse, F)*

For the *government policy and health services related domain*, limited step-down care options available in the community was the main contributing factor expressed by participants that affected discharge planning. This was further complicated by the difficulties in finding foreign domestic helpers due to border control measures or lack of informal caregiving in patients estranged from their families. Thus, discharge was significantly delayed for patients without means to ensure continued care in the community.

*“During lockdown, for the criteria towards day care, you have to be a single elderly, so if you have family, you’re not eligible. But a lot of times, the family is not supportive, or not every family has a fully equipped caregiver. So they rely on day care service so that the patient can attend to, basically can discharge to the community. I think these are the factors that delay care transition.” (#P037, social worker, F)*

However, even for those who had been offered step-down care options after the restrictions were eased, there seemed to have been considerable waiting time in the initiation of community rehabilitation, resulting in overstay in the community hospital.

*“Someone from the Agency of Integrated Care called me just after my mum’s discharge [from community hospital] and asked whether I’m interested in the day rehab… to replace the physiotherapy. But then she has been on the waiting list for almost 3 months.”* (#P020, caregiver, F)

Many also felt that alternative services offered in place of community services such as home exercise did not adequately meet the functional needs of some patients, which led to less-than-optimal recovery.

*“In a way, it [home exercise programme] helps. But I think it is still different from having a professional train you, which is what you would get in a Day Rehab Centre. Because of certain safety restrictions, the type of activities that we prescribe the patient for the home exercise programme may not be sufficient to maximise their potential.” (#P040, therapist, F)*

Lastly, owing to the rapidly evolving situations of the pandemic, there appeared to be limited communication between government bodies and HCWs on the ground. Several HCW participants reported insufficient communication of information and instructions between authorities and HCWs. Consequently, some HCWs had to exercise their own discretion in the course of operating services, evoking feelings of confusion and frustration.

*“It was quite stressful because most of the time I felt that I was searching for new information or instructions myself or my other friends in the social work sector sent me pdf versions [of the new instruction] on WhatsApp, so that was something I felt it became your own personal responsibility to keep being updated yourself.” (#P019, medical social worker, F)*

As expected, this affected communication with patients and caregivers downstream, who felt stressed over the lack of information on caregiving.

### Perceptions of essential services for care integration during lockdown

We explored perceptions of major community services which had been halted during the lockdown and hence potentially affected timely discharge planning. Many HCW and caregiver participants commonly maintained that certain services should have been classified as ‘essential’, such as Day Rehab Centres and Day Care Centres, despite lockdown. These services were seen as critical to motivate patients to stay the course for rehabilitation and to enable them to regain functional ability to perform everyday tasks, thus minimising caregiver burdens.

*“I think what should have continued is rehab services. Rehab is discontinued so now she is not able to walk like the way we trained her, we felt that her condition deteriorated, and her muscles became weaker so then she also lost confidence. We were not very sure whether we were doing it right. I feel very depressed. And there was no one to ask for help.” (#P049, caregiver, F)*

It was also commonly viewed that these services would promote the mental wellbeing of older patients, particularly during the times of social isolation and distress brought on due to lockdown. As one participant noted, the absence of participation in meaningful activities created negative consequences such as depression and, for some, a wish to die.

*“I think one of my residents because he used to be attending senior care centre every day and now because of COVID, he couldn’t go there anymore, so he actually had suicidal ideation, because he felt very bored and depressed at home.”* (#P013, nurse, female)

Another service that participants felt was vital was case management and cluster support (i.e., assessment of holistic care needs and coordinate services). HCWs believed that case management could act as a crucial “safety net” to check in on older adults transitioned to the community, especially those who live alone.

*“Case management is usually offered to patients who are more vulnerable to the detrimental effects of the service disruptions…So if they do stop the case management, then I think it’s very hard for this group of people to survive alone out there. Typically, these services are also a safety net that we set up before they were discharged.” (# P040, therapist, F)*

Some participants maintained that Medical Escort and Transport services should have been essential for frail older adults to ensure continuity of treatment. Both HCWs and patients also noted that home modifications were vital for timely discharge and safe management of patients at home. As one therapist stated, suspension of home modification services during lockdown posed significant impediments to discharge planning:

*“Some patients require a certain equipment or environmental modification to be home, because of lockdown, we had no choice but to wait for that to be ready before we can safely discharge patients.”* (#P040, therapist, F)

On the other end of the spectrum, government officials reflected that closure or restriction of such community services was made after careful deliberation, with the risk of COVID-19 transmission outweighing any detriments to patients. This view was echoed by a minority of HCWs who understood the necessity of closure of the community services during the lockdown in order to limit the transmission of COVID-19. Government officials commonly indicated the difficulty balancing the extent of certain restrictions in the name of public safety and catering to what other stakeholders viewed as equally pressing needs.

*“For the long-term care setting, the definition, I guess, essential is a bit of a loaded word. I wouldn’t say that they are non-essential per se but that because of the concerns about safe distancing and exposure to community risk, we felt that it is safer for seniors to remain at home rather than to go for centre-based activities where they may be exposed to a larger group of people.” (#P043, government official, M)*

### Ways to improve seamless transition of care

Participants made some suggestions on how care transition could be improved for older adults in the event of future public health emergencies. They can be classified into three domains in terms of what each stakeholder group could do: patient and caregiver related; HCW related; and government policy and health services related.

For the *patient and caregiver related domain*, both HCWs and caregivers recognised the utility of telehealth as a way to empower older patients and foster delivery of seamless transition of care. Suggestions were made in terms of the provision of technical equipment, education and awareness of digital literacy, assistance from community health centres. HCWs further emphasised the importance of ‘kampong spirit’ (a sense of community and solidarity), where patient care can be shared by the community and neighbours.

*“Definitely empowering the elderly… the vulnerable elderly and able-bodied elderly, you know their neighbours, to have this community kampong spirit if they can sort of look out for one another, that would not be so dependent on one nurse to do it. But definitely is to empower them to know where to seek help.” (#P022, doctor, F)*

*“Community support is very important, it just doesn’t end at the Community Hospital or discharge, there needs to be a downward stream of care given to the patients because not everybody is so blessed with family to help, sometimes seniors just live alone.” (#P032, therapist, M)*

Greater social interaction and community engagement were suggested by many caregiver participants to address diminished social interaction and mental wellbeing during difficult times.

*“I suppose a listening ear would help. So that we can share our problems, then because of their experience, they could offer us some suggestions as to how we could have gone about doing it. Minimise our effort and our stress level, because that time was really very stressful.” (#P049, caregiver, F)*

Under the *HCW-related domain*, participants commonly suggested that effective communication among HCWs across hospitals and communities through enhanced relationship building and partnership would be essential to improving discharge planning and care continuity.

*“I feel that communication between all the essential service and all the community partners is important to support the elderly patients especially those that are isolated, staying alone.” (#P046, community partner, F)*

It was also stated that having a positive outlook and improving adaptability in care professionals would be crucial in order to respond to unexpected challenges and risks associated with future pandemics.

*“You have to be quick in your response as providers. I think to survive and to be sustainable, you have to be very adaptive. Whatever that happens, take it as a learning experience, and we strive to make it a positive learning experience for us” (#P044, community partner, F)*

For the *government policy and health services related domain*, participants suggested communication of clear guidelines pertaining to centre operating hours and nature of restrictions in a more efficient manner to HCWs.

*“Perhaps early notification of any changes that are to come will help. Because the situation is so fluid and things change quite fast, sometimes I think our advisory may not give them a lot of leeway or advance notice, because we ourselves are also rushing, not that we are taking our own sweet time. So that is one, to give them [healthcare workers] ample notice.” (#P030, Government official, F)*

Some HCWs and community partners also opined that the classification of community services determined essential or non-essential during the lockdown could be revisited for a future public health crisis to ensure that important services remain available for certain vulnerable older adults, albeit with public health measures in place.

*“I think instead of complete closure, there should be some allowance to continue to take, in case of patients who really need to come in, based on a case-by-case assessment. So that there should still be options for the caregivers, even if it’s reduced frequency…and we also can limit to certain numbers.” (#P033, community partner, F)*

Setting up of contingency care models were suggested where services could be continued with minimal disruptions in the event of another pandemic, such as a ready pool of manpower from private sectors that can be mobilised during times of need. Another suggestion made by both caregiver and HCW participants was the streamlining of services, which could be achieved through sharing of patient information across care providers in the community and having a single provider to support all the patient’s home-based needs to minimise time spent on handovers and to foster a greater transition of care.

## Discussion

This study sheds light on the impact of the COVID-19 measures on discharge planning and continuity of care in the community through the views and experiences of multi-stakeholders.

Our study found that discharge planning and continuity of integrated care in the community were considerably affected by a range of factors during the lockdown: the reluctance of family members to accept community services due to the fear of COVID-19 infection, patient’s lack of digital literacy, challenges of remote assessment by HCWs for post-discharge care, manpower shortage, limited step-down care options and lack of clear formal guidelines. These factors may have led to delayed discharge, and the effect seemed to be disproportionately acute for older patients who live alone without family support. This finding resonates with prior literature that the absence of care options for vulnerable patients accounted for considerable delay in hospital discharge [7,24], and that poor communication during the delayed care transition further exacerbated the feelings of uncertainty [7]. However, our finding is in contrast with a recent COVID study that found a significantly decreased length of hospital stays among patients with low functional status, despite limited community resources [15]. This could possibly be explained by patients’ preferences for early discharge for fear of infection albeit limited community resources or heightened efforts by healthcare institutions to free up available beds for COVID-19 patients. There might have been other unmeasured factors such as patient case-mix in the study which may explain the decreased length of hospital stay. Prolonged hospital stay was found to be associated with an increased risk of nosocomial infections and significant functional decline [25]. We propose a business contingency plan where continuity of integrated care can be ensured for vulnerable patients, through novel means such as telehealth. Adequate bidirectional communication of information between the authorities and HCWs regarding services that are available and the recommended discharge plans could also improve the care transition. Promotion of bidirectional communication would also allow HCWs an opportunity to close gaps in understanding that may not be apparent in government officials and build mutual trust between HCWs and the authorities. Empowering patients to better navigate access to services as well as garnering support from the neighbours and community partners to enable shared responsibility of patient care could also improve continuity of care in the community as suggested by our participants. Stronger neighbourhood networks are found to mitigate poor health outcomes for vulnerable older patients in the community during the lockdown [26]. Therefore, it would be important to strengthen neighbourhood communities to ensure the wellbeing of older patients discharged to the community.

Another key finding was the perceived essentialness of community services for a smooth transition of care amidst the COVID-19 pandemic. Many participants believed that certain community services, such as day rehab, cluster support and home modification, should have been provided in a safe manner as they witnessed significant delays in discharge with negative implications. Some community services were commonly viewed by participants as not only for promoting rehabilitation, but they were the sites of social interaction for older adults [27]. Indeed, prior literature suggests that participation in rehabilitation and daycare centres has been shown to reduce depression and improve quality of life in older adults [27,28]. Community Day Care Centres can also decrease caregiver burden and help caregivers cope with demands by providing much-needed respite and support services [29]. It has been suggested that loss of social interaction and physical inactivity during lockdown result in a decline in mental and physical health among vulnerable older populations [30]. Likewise, greater social isolation is associated with higher depressive symptoms and anxiety [31]. However, whilst continuity of community services could benefit older adults and their caregivers, the risk of viral transmission in such settings cannot be underestimated, as stated by the government officials in our study. Although the risk of COVID-19 transmission in community services is not well studied, the pandemic has disproportionately affected long-term care facilities such as nursing homes and rehabilitation facilities, with massive outbreaks being reported worldwide [32,33]. In addition, people with chronic comorbidity and older adults are found to be more susceptible to the infection due to weakened immunity [34]. In view of these considerations, it is important to revisit the definition of essential services through the engagement of stakeholders including HCWs, social service providers and frontliners who work closely with patients to arrive at consensus, while weighing against the risk of COVID-19 infection. Formation of a pandemic taskforce should also consider including these stakeholders to better understand the issues on the ground.

Our findings underscore the importance of novel models of care in the era of restricted in-person health care. Telehealth, including phone and video consultation, was tried, and tested in the community to enhance care accessibility for older patients. Telehealth allowed for continued care while reducing exposure to infections, a care model instrumental for routine monitoring and support for older adults with multiple chronic conditions following hospital discharge. A study found no difference in mortality for patients receiving telehealth as compared to in-person care for diabetes and congestive heart failure [35], while other studies found that telehealth reduced hospital readmissions in patients with heart failure [36,37]. Despite apparent benefits of telehealth, our study found a myriad of challenges in the use of telehealth for these vulnerable older adults, including limited digital literacy to utilise technology, hearing impairment or failure to fully describe symptoms and care needs. Such challenges may hinder the ability of telehealth to adequately replace face-to-face care provision for older patients. This is echoed in a study where age, level of education and computer literacy were the key patient-related barriers to telehealth [38]. In light of these challenges, it is an opportune time to step up efforts to assess the level of digital literacy in older patients, address concerns they might have and educate them on using digital devices. When building novel models of care including telehealth, it is also important to integrate the digital platform into the IT ecosystem so that information sharing and communication across care providers can be enhanced. [39] Suboptimal care coordination across different services should be addressed through a shared effort to realise a better care model. Participants’ suggestion on having a single provider to support patients’ home-based needs clearly warrants further exploration and discussion.

### Strengths and Limitations

To our knowledge, this is the first study that provides a nuanced understanding of the impacts of COVID-19 measures on discharge planning and continuity of care in the community for older adults with long-term chronic conditions. Notwithstanding the strengths, our findings should be considered in light of a few limitations. Despite efforts to involve more patients and caregivers, we had a lower response rate from this group. In particular, the voice of patients as end users was notably absent from the interview pool of stakeholders; recruiting patients who were well enough to take part in the interview was exceptionally difficult during the lockdown. It is possible that this might have influenced the findings. However, we ensured the inclusion of a wide range of HCWs and other stakeholders, which allowed for cross-validation of responses and triangulation of consensus views of the impacts of COVID-19 measures on the experience of care transition. A further limitation is that our sample was skewed towards female participants. Although this may be a limiting factor, it is important to note the predominance of females in healthcare workforce and informal caregiving in Singapore. Remote interviewing via video conferencing might have excluded some caregivers and patients with low digital literacy. Lastly, the study only assessed the immediate impacts of the COVID-19 measures on discharge planning and care continuity. Longitudinal research is needed to explore the long-term effects of the measures.

## Conclusion

The COVID-19 measures had a significant impact on safe and timely discharge of vulnerable older patients and care continuity in the community. Findings illuminate the need to proactively assess care pathways and catalyse novel models to improve care transition. Better communication between HCWs and relevant authorities and between care providers across sectors is needed to improve the coordination of care. The essentialness of community services amidst pandemics may need to be revisited to address the needs of vulnerable older patients. Garnering support from the community to enable shared responsibility of patient care could also improve continuity of care.
